# Editorial: Case reports in hematological malignancies: 2022

**DOI:** 10.3389/fonc.2023.1272547

**Published:** 2023-08-21

**Authors:** Ahmad Antar, Arpad Szallasi, Osamu Imataki

**Affiliations:** ^1^Department of Hematology-Oncology, Almoosa Specialist Hospital, Al-Ahsa, Saudi Arabia; ^2^Faculty of Medicine, Semmelweis University, Budapest, Hungary; ^3^Faculty of Medicine, Kagawa University, Kita-gun, Japan

**Keywords:** hematological malignances, leukemia, lymphoma, multiple myeloma, myeloproliferative neoplasms, bone marrow transplantation (BMT), CAR (chimeric antigen receptor) T cells, case reports

Hematological malignancies are tumors of the hematopoietic and lymphoid tissues, referring to a group of cancers that affect the blood, lymph nodes, and bone marrow. They include leukemias, myelodysplastic syndrome (MDS), myeloproliferative neoplasms (MPNs), lymphomas, plasma cell neoplasms, and other rare bone marrow diseases like histiocytic or dendritic cell tumors. The most recent World Health Organization (WHO) classification of hematolymphoid tumors (5^th^ edition) was released in August 2022 (1).

The aim of this Research Topic entitled “*Case reports in hematological malignancies*” was to collect articles that highlight rare cases with typical features, frequent cases with atypical features, or cases with a convincing clinical response to new off-label use therapy. Such cases may provide valuable insights into the pathomechanism of the disease. They can also serve as a basis for creating hypotheses for future research. A total of seventy-three manuscripts were submitted, of which thirty-three have been accepted for publication: a 45% acceptance rate.

Eleven of the accepted manuscripts are cases of acute leukemia. Long et al. reported a case of acute myeloid leukemia (AML) treated with a lower dose of venetoclax (100 mg once daily) with the combination of grapefruit juice (a strong CYP3A4 inhibitor, 200 ml 3 times daily), and azacitidine 75 mg/m2 on days 1–7 of each 28-day cycle (2, 3). The patient achieved complete remission (CR) with the venetoclax Cmax within the effective concentration range at 7 and 14 days of treatment and maintained remission with manageable side effects. This case highlights the importance of drug-food interaction combinations in reducing the dose and cost of venetoclax, especially in low to middle-income countries. A case of concurrent Langerhans cell histiocytosis (LCH) and AML which shared driver mutations in an 84-year-old female patient was reported by Kazama et al. The diagnosis was based on biopsy where LCH was primarily identified in the skin, lymph nodes, and bone marrow, while AML was predominantly present in the bone marrow and peripheral blood. Only a few numbers of cases of LCH and AML diagnosis at the same time have been reported, the majority of which included generalized LCH, and monocytic leukemia, and was associated with dismal prognosis (4, 5). In summary, this case is in favor of classifying LCH and other forms of histiocytosis as myeloid/myeloproliferative malignancies.

Two cases of invasive fungal infection in AML patients were reported. Tabak et al. described a case of AML with lungs and sinuses mucormycosis at diagnosis and who had a loss of function mutation in the *TNFRSF13B* gene. This mutation increases the likelihood of developing immunodeficiency syndromes since it has been demonstrated to impair B-cell differentiation and homeostasis (6). These data highlight the importance of next-generation sequencing for the prognosis and treatment of AML. The other case was a patient with myeloid sarcoma in her right breast who, while receiving the first consolidation course of chemotherapy, acquired a severe fungal infection caused by Saprochaete clavata. The patient was refractory to maximum antifungal therapy and the treatment was successful only when granulocyte transfusion therapy was initiated. In this case, Pasqualone et al. suggest the addition of granulocyte transfusions as adjunctive therapy for patients with profound and long-lasting neutropenia and severe fungal infections resistant to broad-spectrum antimicrobial treatment (7).

Interestingly, three cases of atypical acute promyelocytic leukemia (APL) were presented in this Research Topic. Zhao, J. et al. reported a case of AML with Retinoic acid receptor gamma gene rearrangement which resembled classical APL in clinical features, morphology, and immunophenotype but did not carry the diagnostic PML-RARA fusion gene (8). This APL-like case showed resistance to all-trans retinoic acid (ATRA) and Arsenic trioxide but responded well to homoharringtonine and cytarabine. The second case reported by Chen, L. et al. was a variant APL with BCOR-RARA instead of PML-RARA fusion gene. The patient was successfully treated by ATRA plus standard chemotherapy followed by allogeneic hematopoietic stem cell transplantation (allo-HSCT) and ATRA maintenance and maintained molecular remission. Variant APL cases have a high risk of relapse which warrants accurate identification and optimal aggressive therapy (9). The third case reported by Chen, X. et al. was an APL-like case in a 3-year-old child caused by torque teno mini virus (TTMV) fragment integration into the RARA locus which suggests that TTMV: RARA is a recurrent cause of APL missing PML: RARA. Only two similar APL-like cases caused by TTMV integration were reported in the literature (10, 11). This case highlights the identification of more TTMV: RARA positive APL-like cases considering the widespread prevalence of TTMV in the general population.

Two reports of stem-cell transplantation in AML patients were included in this Research Topic. Zheng et al. reported the first 2 cases in the literature of successful haploidentical allo-HSCT in AML patients from donors having mild alpha(a)-thalassemia. Follow-up on these 2 patients showed a full donor chimerism, no development of chronic GVHD, and both experienced conversion to donors’ thalassemia type with mild microcytic anemia and maintaining Hb levels between 10 to 12 g/dL without transfusion.

Wang, Y. et al. reported a case of Gastrointestinal-GVHD in an AML young female after allo-HSCT that was complicated by small bowel obstruction. The patient experienced prolonged bloody diarrhea after stopping cyclosporine and the gradual reduction in steroids. Imaging showed intestinal stricture 10 months after the transplant. She was treated successfully with surgical resection of the small intestinal stenotic loop after the failure of appropriate immunosuppressive therapies.

The last two case reports in the acute leukemia set were two cases of acute lymphoblastic leukemia (ALL). Ni et al. reported a case of Donor-derived leukemia (DDL) in a patient who was initially diagnosed with B-cell ALL, for which she received induction chemotherapy then she received salvage chemotherapy after relapse. Last she underwent umbilical cord blood transplantation (UCBT) after the first relapse and achieved CR2.

Unfortunately, the patient had a disease relapse again after UCBT. Interestingly, the result of chimerism still showed complete donor chimerism which is consistent with DDL. DDL is an uncommon but serious complication after transplant (12). DDL after UCBT is usually resistant to chemotherapy and the prognosis is very dismal (13). The patient was resistant to chemotherapy, however, she received anti-CD19 chimeric antigen receptor T-cell (CAR-T) cell therapy and achieved CR with negative minimal residual disease (MRD). Then she received preemptive interferon-a treatment after the rise of MRD levels and maintained CR for 41 months. Interferon-a enhances the graft-versus-leukemia effect and is used as an adjuvant or maintenance therapy after transplant to eliminate MRD and lower the risk of leukemia recurrence (14). This study offers a novel therapeutic strategy for DDL.

The second case reported by Wang, R. et al. was a child with B-cell ALL carrying the rare mutation TP53 c.C275T. This mutation is very rare and is associated with poor prognosis (15). The patient showed resistance to most conventional chemotherapy regimens. *In vitro*, drug sensitivity tests have shown that bortezomib had a very strong susceptibility to the patient’s leukemic cells. Therefore, the patient was then treated with bortezomib in combination with vindesine, fludarabine, and cytarabine. She achieved MRD-negative CR after one course of therapy and continued recurrence-free after a 9-month. According to this report, bortezomib in combination with chemotherapy may be an effective therapy for ALL patients carrying TP53 c.C275T mutation.

Cases of chronic leukemias are well represented in this Research Topic. Ramdohr et al. reported 2 patients with atypical chronic myeloid leukemia (CML) carrying the BCR: ABL1 e6a2 fusion transcript. Most CML patients are diagnosed with the e13a2 or e14a2 BCR: ABL1 fusion transcripts. However, there is a growing number of CML cases with atypical clinical presentations that are associated with other transcripts, such as e19a2, e8a2, e13a3, e14a3, e1a3, and e6a2. These transcripts have been described in 1% of CML patients and their clinical significance is yet to be determined (16–18). For example, the atypical e6a2 BCR: ABL1 transcript seems to portend poor prognosis as it is associated with an aggressive phenotype and early transformation into acute leukemia (19). The reported 2 patients expressing the atypical e6a2 BCR: ABL1 fusion transcript were treated effectively with the 2^nd^ generation tyrosine kinase inhibitor, nilotinib. The second article on CML was reported by Pasquale et al. The patient developed severe pleural effusion secondary to nilotinib therapy and was managed with steroids and permanent withdrawal of the medication. Pleural effusion is a well-known toxicity related to dasatinib, which has been linked to either the expansion of cytotoxic T and NK cells or the activation of additional kinases (20). But it’s extremely rare during nilotinib therapy, especially in first-line settings.

Bi et al. described the first case of the coexistence of primary pulmonary lymphoma and opportunistic pneumonia in a patient with CML on imatinib therapy. Primary pulmonary lymphoma is a rare neoplasm and has very nonspecific clinical features that can overlap with infection and often cause delay or neglect in diagnosis (21). This patient continued to have lung disease progression even after optimal antimicrobial coverage. The lymphoma was then diagnosed by CT-guided biopsy, and he received chemotherapy and is still alive at the date of report writing.

Vermeersch et al. reported a case series (n=5) of chronic neutrophilic leukemia (CNL) associated with monoclonal gammopathy. They analyzed the genetic characteristics of these cases using cytogenetic and molecular studies. CNL is a rare type of BCR: ABL1 negative MPN, defined by persistent mature neutrophilia and hypercellular bone marrow (22). CSF3R mutation is found in up to 80% of CNLs. CNL is associated with plasma cell neoplasms in approximately 32% of cases, wherein CSF3R mutation tends to be less frequent (23, 24). In this series, 3 patients have shown a predominant lambda light chain expression. Four patients progressed into plasma cell myeloma. All patients received treatment for the associated malignancies such as AML and multiple myeloma (MM), and among these two patients underwent allo-HSCT.

Two manuscripts reported cases of chronic lymphocytic leukemia (CLL). The first one by Valvano et al. described a very rare association of CLL with hairy cell leukemia (HCL) with the challenges in diagnosis using multicolor flow cytometry and PCR (25). The patient was initially treated with cladribine and rituximab. The other report by Ballotta et al. described 2 cases of CLL with post-COVID-19 condition. Both patients had persistent SARS-CoV-2 infection without seroconversion for 7-8 months. The patients were effectively treated with monoclonal antibodies casirivimab/imdevimab infusion. This data highlights the effectiveness of monoclonal antibodies in immunocompromised patients with persistent SARS-CoV-2 infection (26).

The topic of lymphomas with rare presentation has been covered in 10 articles. Among them, 3 reports presented the unusual association of lymphoma with other neoplasms. A case of concurrent diffuse large B-cell lymphoma (DLBCL), rectal adenocarcinoma, and hepatocellular carcinoma was reported by Qiu et al. He was successfully treated with six cycles R-GEMOX followed by six cycles R-CHOP to achieve CR for DLBCL. During this course of therapy, the patient received a PD-1 inhibitor (Sintilimab) as a treatment for HCC and, ultimately, he ended up with curative intent right hemihepatectomy and Dixon surgery. The second case reported by Bhakta et al. described an elderly man with untreated prostate cancer who presented with left thigh pain and eventually developed a pathologic fracture. After fixation surgery, the pathology came out as DLBCL. Staging by PET scan FDG revealed no evidence of disease outside the thigh. The patient was then diagnosed with primary bone lymphoma (PBL) and was treated with chemotherapy R-mini-CHOP protocol. PBL represents less than 5% of all primary bone malignancies and less than 1% of all lymphomas (27). Pathologic fracture is an unusual complication of PBL (28). The authors highlight the importance of suspecting PBL in patients with pathologic fractures. The third case was reported by Accorsi Buttini et al. who described the development of high-risk MDS following CAR T-cell therapy in a patient with relapsed refractory DLBCL. Prolonged pancytopenia after CAR T-cell therapy is most likely related to active inflammation milieu together with the impact of previous cytoreductive chemotherapy (29). Bone marrow biopsy showed deletion of chromosome 7 and acquisition of RUNX1 mutation. The authors concluded that the development of MDS could be related to previous chemotherapy, however, impairment of immunosurveillance related to either lymphodepletion or CAR T-cell infusion could play a role too. Two case reports of lymphoma with unusual site involvement were described in this Research Topic. Johnson et al. reported a case of classic HCL with CNS involvement at relapse. The patient was initially treated with cladribine, pentostatin, and rituximab with long-lasting response till he developed CNS disease. Most classic HCL harboring BRAF V600E mutation offers a targeted therapy for the pretreated population (30). This patient achieved CR of relapsed HCL with CNS involvement after treatment with a BRAF inhibitor (Vemurafenib). Yang et al. reported a case of relapsed/refractory DLBCL with cardiac involvement. The patient developed cardiac metastasis after 12 months of treatment by conventional first-line chemotherapy and anti-CD19 CAR T cell immunotherapy. Secondary cardiac lymphoma is a rare disease and is associated with a high mortality rate (31). The patient was then treated with salvage chemotherapy, followed by CAR-NK cell immunotherapy and allo-HSCT. Unfortunately, he died of severe pneumonia. This report highlights the significance of early diagnosis and timely treatment to improve the prognosis of secondary cardiac lymphoma.

Two articles on Burkitt Lymphoma (BL) and CAR T-cell therapy have been presented in this Research Topic. Ye et al. reported a 61-year-old patient with high-risk relapsed-refractory BL who was treated successfully by salvage auto-HSCT followed by CAR T-cell consolidation therapy. The authors highlight the safety and feasibility of CAR T-cell therapy for older high-risk BL patients. The patient is still in CR after 4 years of treatment. The second case, by Wang, Y.-L. et al., is a young patient who developed cognitive impairment after CAR T-cell salvage therapy for BL. Neurotoxicity, including cognitive impairment, is a well-known adverse event of CAR-T cell infusion and is associated with a poor outcome (32). The main mechanism of neurotoxicity is neuroinflammation secondary to cytokine release syndrome and effector cell-associated neurotoxicity syndrome (33). Currently, CAR-T therapy-related cognitive impairment has no effective treatment. Sodium oligomannate is an effective treatment for cognitive deficits in mild-to-moderate Alzheimer’s dementia through inhibition of neuroinflammation (34). The patient was treated with sodium oligomannate with rivastigmine and had significant improvement in cognitive function.

Sun et al. reported a case of lymphoma-associated hemophagocytic lymphohistiocytosis complicated by acute necrotizing encephalopathy. This complication is related to cytokine storm. Brain insult secondary to this disease is not uncommon, however, the association with acute necrotizing encephalopathy is rarely reported (35). The patient was successfully treated with chemotherapy, immunoglobulin, and early initiation of steroids.

One case of lymphoma with pathologic challenges was reported by Zhang et al. The patient was diagnosed with AITL, or Angioimmunoblastic T-cell lymphoma. AITL is always presented with immense follicular dendritic cell meshwork (36). In this case, the proliferation of spindle cells was so profuse that was easily misdiagnosed as follicular dendritic cells sarcoma. Immunochemical and molecular examinations were consistent with AITL.

The last article about lymphoma was reported by Xing et al. The patient was diagnosed with double expression (MYC and BCL2) DLBCL with mutations of ATM and CD58 genes. She received 4 cycles of R-CHOP plus zanubrutinib and achieved only a partial response. An *in-vitro* high-throughput drug screening using a panel of 117 compounds was applied and showed sensitivity to single-agent bortezomib, thalidomide, and gemcitabine, and to the combination of bortezomib, thalidomide, and dexamethasone (VTD). The patient achieved CR after 2 cycles of VTD followed by 2 cycles of VTD-gemcitabine.

Moving to plasma cell neoplasm case reports, Bonometti et al. reported a case of small bowel obstruction secondary to plasma cell myeloma (PCM). GI involvement by PCM is extremely rare (37). The patient presented with abdominal pain and signs of small bowel obstruction by CT scan abdomen, for which he underwent urgent ileal resection. Histopathology revealed PCM. Subsequent investigations found serum monoclonal gammopathy, osteolytic bony lesions, and clonal bone marrow plasma cell infiltrate. He was treated with myeloma protocol. The authors emphasize the importance of prompt diagnosis and management of rare GI presentation of PCM. Another case of rare extramedullary disease of myeloma was reported by Li et al. They presented a patient with isolated CNS relapse 7 years after being treated for myeloma by induction therapy followed by auto-HSCT. CNS involvement is very rare, it occurs in only about 1% of patients (38). Isolated CNS relapse is even rarer with only a few case reports, these patients have a very poor prognosis (39). The patient was treated successfully with high-dose methotrexate and lenalidomide. Otsuka-Kamakura et al. reported a case of plasmablastic neoplasm with multinucleated giant cells that were difficult to distinguish from plasmablastic lymphoma or PCM of plasmablastic type. Both multinucleated giant cells and mononuclear cells had the same profile, according to IHC and FISH analyses. The authors concluded that the multinucleated giant cells were compatible with cancer stem cells.

The last three published articles in this Research Topic presented rare hematological diseases. Yoshida et al. reported a case of Rosai-Dorfman disease (RDD) with simultaneous multiple extranodal lesions in the skin, heart, liver, and pelvis. The patient has no cervical lymphadenopathy. She was successfully treated with steroids. RDD is a non-Langerhans cell histiocytosis characterized by fever and bilateral cervical lymphadenopathy. It’s commonly presented with extranodal site lesions in the skin, bones, head and neck, kidneys, and CNS (40). The simultaneous cardiac and pelvis involvement has not been reported. This case highlights the atypical distribution of uncommon extranodal lesions of RDD and the need to avoid delays in diagnosis and treatment. Xu et al. reported three cases of TEMPI syndrome. TEMPI syndrome is a novel and ultra-rare disease characterized by Monoclonal gammopathy, Erythrocytosis, Telangiectasis, Perinephric fluids collection, and Intrapulmonary shunting (41). Only 29 cases were reported worldwide (42). The patients showed various responses to Myeloma-directed therapy. The response to therapy was assessed by measuring hemoglobin, erythropoietin, and M protein levels throughout the course of treatment. Zhao, L. et al. reported 2 cases of HLH, the first one was secondary to NK T-cell lymphoma and the second one was associated with missense variants in the perforin 1 gene. Both cases responded rapidly to ruxolitinib plus dexamethasone protocol without obvious adverse effects. The primary first-line treatment for HLH is chemotherapy-containing regimens. However, some patients are resistant to treatment or unfit for intensive chemotherapy (43, 44). This report highlights the importance of ruxolitinib plus dexamethasone as a potential treatment for HLH.

In summary, the thirty-three articles published on this Research Topic provide clinical practitioners and researchers valuable insights into diagnosing and treating diseases with unusual presentation and open the door for future research.

## Author contributions

AA: Writing – original draft, Writing – review & editing. AS: Writing – review & editing. OI: Writing – review & editing.
